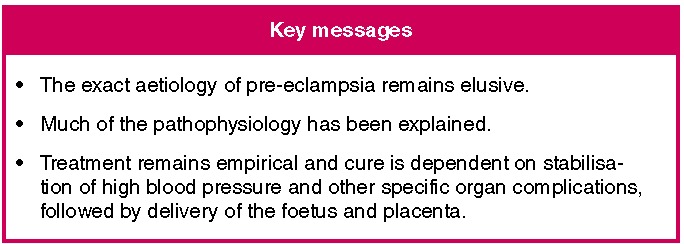# Pre-eclampsia: its pathogenesis and pathophysiolgy

**DOI:** 10.5830/CVJA-2016-009

**Published:** 2016

**Authors:** P Gathiram, J Moodley

**Affiliations:** Department of Physiology, Women’s Health and HIV Research Group, Nelson R Mandela School of Medicine, University of KwaZulu-Natal, Durban, South Africa; Department of Obstetrics and Gynaecology and Women’s Health and HIV Research Group, Nelson R Mandela School of Medicine, University of KwaZulu-Natal, Durban, South Africa

**Keywords:** pre-eclampsia, major cause of maternal mortality and morbidity, placenta

## Abstract

Pre-eclampsia is a pregnancy-specific disorder that has a worldwide prevalence of 5–8%. It is one of the main causes of maternal and perinatal morbidity and mortality globally and accounts for 50 000–60 00 deaths annually, with a predominance in the low- and middle-income countries. It is a multisystemic disorder however its aetiology, pathogenesis and pathophysiology are poorly understood. Recently it has been postulated that it is a two-stage disease with an imbalance between angiogenic and anti-antigenic factors. This review covers the latest thoughts on the pathogenesis and pathology of pre-eclampsia. The central hypothesis is that pre-eclampsia results from defective spiral artery remodelling, leading to cellular ischaemia in the placenta, which in turn results in an imbalance between anti-angiogenic and pro-angiogenic factors. This imbalance in favour of anti-angiogenic factors leads to widespread endothelial dysfunction, affecting all the maternal organ systems. In addition, there is foetal growth restriction (FGR). The exact aetiology remains elusive.

## Abstract

Pre-eclampsia (PE) is a disorder of pregnancy with a worldwide prevalence of about 5–8%. It is characterised by new-onset hypertension with systolic blood pressure ≥ 140 mmHg or diastolic blood pressure ≥ 90 mmHg, measured on two occasions at least four hours apart, and proteinuria of > 0.3 g per 24 hours or ≥ 1+ proteinuria, detected by urine dipstick after 20 weeks of pregnancy, or in the absence of proteinuria, new-onset hypertension with new onset of any one of the following: thrombocytopaenia (platelet count < 100 000/μl), renal insufficiency (serum creatinine concentration > 1.1 mmg/dl or a doubling of the serum creatinine concentration in the absence of other renal disease), impaired liver function (raised concentrations of liver transaminases to twice normal concentrations), pulmonary oedema, or cerebral or visual problems.^1^

PE is one of the main causes of maternal mortality, resulting in about 50 000–60 000 deaths annually worldwide.^1^ In addition, it is associated with an increased risk of the mother and her child developing cardiovascular complications and diabetes mellitus later in life.^2^ Furthermore, PE is a multi-systemic syndrome, involving genetic and environmental factors in its pathogenesis and pathophysiology and the only known treatment is delivery of the foetus and placenta.^3^

In addition, there are subtypes of PE, which are based on the time of onset or recognition of the disease. It is generally divided into two main types, early- and late-onset PE.^4^ The latter comprises the majority (> 80%) of pre-eclamptics. In the earlyonset type, the clinical signs appear before 33 gestational weeks, while in the late-onset type they occur at and after 34 weeks. However, it is the early-onset type that is responsible for most of the high maternal and foetal mortality and morbidity rates.

The main pathological feature of early-onset PE is incomplete transformation of the spiral arteries, resulting in hypoperfusion of the placenta and reduced nutrient supply to the foetus. This results in signs of foetal growth restriction (FGR).^5^ On the other hand, in late-onset type, the spiral arteries, if at all, are slightly altered in diameter and there are no signs of FGR.^6^ This is because early-onset pre-eclampsia is related to placental hypoperfusion, while in the late-onset type there is either no change or a shallow modification of the spiral arteries, leading in some cases to hyperperfusion of the placenta.^7^-^9^ Therefore, it seems that early- and late-onset PE have different pathophysiological and aetiological pathways.^9^

In normal pregnancy, the extracellular fluid and plasma volumes increase by 30–50% and 30–40%, respectively, perhaps due to a decrease in systemic vascular resistance and increase in cardiac output.^10^ The decreased systemic vascular resistance is thought to be due the presence of nitric oxide.^11^ In early-onset PE, there is a decrease in plasma volume, occurring at 14–17 gestational weeks,^9^ before the clinical onset of the disorder.^12^ This aspect is discussed more fully later under the role of the renin–angiotensin–aldosterone system (RAAS).

Despite decades of research, the pathogenesis and pathophysiology of PE are still poorly or incompletely understood.^4^ The pathogenic process of PE begins during the first trimester, long before clinical signs are apparent. Hence it is difficult to identify early biomarkers. The main reason for this is perhaps ethical in nature, as it is difficult to conduct studies in early pregnancies, as these may compromise both the mother and child, and furthermore the pathogenic processes could be multifactorial. In any event, it is generally felt that lack of adequate placental development is the root cause of early-onset PE because the only known treatment of the disorder is delivery of the foetus and placenta. It is, however, essential to understand the features of placental development in normal pregnancies in order to understand the pathophysiology of PE.

## Role of the placenta in normal pregnancies

Placentation and trophoblast invasion of the maternal tissue involves two processes, firstly vascularisation to establish a foeto-placental vascular network, and secondly, invasion of the maternal spiral arteries by the cytotrophoblasts or endovascular trophoblasts (EVTs).^13^ At the time of implantation, trophoblastic cells differentiate into cytotrophoblasts and syncytiotrophoblasts. The cytotrophoblasts form the extravillous trophoblasts (EVT), which invade the decidual and junctional zone myometrial segments, the inner third of the myometrium and the spiral arteries. The EVTs induce remodelling of the latter, perhaps by causing loss of the elastic lamina, most of the smooth muscle cells, and temporarily replacing the endothelial cells,^13^ thus transforming a high-resistance, low-flow vascular system into a low-resistance, high-flow type, essential for normal foetal growth.^13^,^14^

Therefore, the cytotrophoblasts, epithelial in nature, replace the endothelial cells and in the process, the epithelial-like receptors are replaced with maternal adhesion molecules such as vascular endothelial (VE) cadherin vascular adhesion molecule-1, platelet-endothelial molecule-1, and αVβ3 integrin.^13^ This perhaps accounts for the prevention of foetal rejection. The trophoblasts therefore take on the phenotype of endothelial cells and are in direct contact with maternal blood, but the maternal and foetal blood do not mix.

The syncytiotrophoblasts are multinucleated, line the chorionic villi, and act as an interface between maternal and foetal blood. However, according to Brosens *et al.* (2011),^15^ trophoblast invasion of the spiral arteries is preceded by oedema of the vessel wall, disintegration of the elastic fibres and changes in the smooth muscle layer, leading to a loss of myofibrils.^15^ Hence it is not the generally believed concept that the trophoblastic cells themselves cause disintegration of the elastic fibres and loss of myofibrils. In addition, development of the foetus initially occurs under low oxygen tension and placental perfusion is only from the intervillous space, and unplugging of the maternal spiral arteries occurs at about the 12th gestational week.^16^

The migration of trophoblasts into the spiral arteries is influenced by a number of factors such as cytokines, growth factors, oxygen tension, and the local cellular environment, for example immune cells such as macrophages and decidual/ uterine natural killer (dNK) cells.^17^ The dNK cells are thought to play an important role in regulating placentation but the exact mechanism of action is still unclear.^18^

## Systemic inflammatory response in normal pregnancies

The foetal trophoblast is regarded as an allo-antigen and the mother reacts to this and mounts a sterile, low-grade systemic inflammatory response.^4^,^19^ It is thought that syncytiotrophoblast microparticles (STMBs) detected in the maternal circulation could be the cause.^20^ However, it is known that utero-foetal perfusion only begins towards the end of the first trimester, while increased levels of STMBs in the maternal circulation are detected during the second and third trimesters.^21^ The initial inflammatory response during the first trimester could be due to an interaction between the decidual immune cells and trophoblast cells, and that a secondary inflammatory response during the second and third trimester could be due to syncytiotrophoblast microparticles released into the mother’s vascular system.^21^,^22^

## Placental blood flow in pre-eclampsia and its consequences

In PE, it has almost been established that there is reduced blood flow to the placenta, especially in the early-onset type, because of defective spiral artery remodelling and acute artherosis.^23^,^24^ *In vivo* techniques (magnetic resonance imaging and Doppler low-flow measurements) have confirmed this in early- but not late-onset PE.^7^

In PE the defects in spiral artery remodelling are restricted to the distal segments of the spiral arteries, that is the proximal decidua and the junctional zone (JZ) myometrial segments, and hence the myometrial spiral arteries still have much of their smooth muscle cells and elastic lamina, with absent or partial transformation of the arteries in the JZ myometrial segment.^4^,^15^

The exact mechanism for this is not known but various factors, such as abnormal genetic variations, biology of the trophoblasts or defective trophoblast differentiation acting together with extrinsic factors, such as maternal constitutional factors, action of macrophage defense mechanisms, impaired action of dNK cells and maternal endothelial cells have been advanced.^18^,^23^,^25^ Recently, it has been proposed that proteolytic activity of the different populations of the EVTs could be involved in invasion of the decidua and spiral arteries.^26^

Studies conducted in our laboratories showed that a PE-like syndrome can be produced in a rat model by reducing the placental blood flow through the administration of nitro-L-arginine methyl ester (L-NAME).^27^,^28^ In addition, co-administration of sildenafil citrate, which blocks the action of L-NAME, prevented the PE-like syndrome. Furthermore, we have shown that once the administration of L-NAME is discontinued, the pathophysiology of PE continues until birth of the pups, and thereafter the high blood pressure and proteinuria return to almost normal levels.^29^

The question then arises as to what the effects of the reduced placental blood flow or hypoperfusion on the maternal syndrome, namely hypertension, proteinuria and oedema, are. Is it the reduced blood flow *per se* that triggers events leading to the maternal syndrome, or is it some other factor/s associated with ischaemia?

It is believed that reduced placental blood flow could result in hypoxia of the placenta, which has been suggested as the ultimate cause of PE.^19^,^30^ However, no in vivo measurements of oxygen tension in the intervillous space have been made to claim that hypoxia does occur.^31^ Nevertheless, it is believed that that reduced blood flow or chronic hypoxia on their own are not the direct cause of the placental lesions seen in PE but could be a contributing factor. It has therefore been assumed that the lesions could rather be due to an ischaemia–reperfusion or hypoxia–reoxygenation (HR) type of injury caused by free radicals such as reactive oxygen species (ROS).^31^

Furthermore, it has been speculated that an intermittent type of blood flow occurs in the intervillous space, which could be responsible for the HR type of injury.^31^ To support this, Yung *et al.* in 2014^32^ showed that high levels of activation of unfolded protein-response pathways due to HR damage to the endoplasmic reticulum occurred in placental samples taken from early- but not late-onset PE. Accumulation of aggregates of unfolded protein response (UPR) or misfolded proteins has been observed in PE placentas and it is believed that these may contribute to the pathophysiology of the disorder.^33^ However, no measurements have been made to show that blood flow to the intervillous space is indeed intermittent. We believe that it is the pulsatile nature of blood flow from the spiral arteries that could be responsible for the HR type of injury.

The defective spiral arteries lead to further deterioration in placental perfusion, ischaemia and worsening of the already hypoxic condition seen in normal pregnancies.^10^ The HR damage to the placenta, however, results in increased stress of the syncytiotrophoblasts, causing necrosis, apoptosis and release of excess placental debris (STMBs and vesicles), compared to a normal pregnancy, into the maternal circulation.^34^ In addition, soluble endoglin (sEng) is the extracelluar component of Eng, which is highly expressed in the syncytiotrophoblasts, and shedding of STMBs causes either mechanical disruption or proteolytic cleavage of sEng, and excess amounts of it are present in PE. The details of this are discussed later in this review.

It is therefore believed that placental ischaemia–reperfusion injury is central to the development of PE. In addition to STMBs, pro-inflammatory cytokines, responsible for endothelial dysfunction and increased inflammatory responses, lead to the clinical signs of PE, such as hypertension, proteinuria and thrombotic micro-angiopathy, presenting as haemolysis, elevated liver enzymes and low platelet count (HELLP) syndrome, pulmonary or cerebral oedema and seizures.^35^,^36^ However, there is no clear evidence that this really occurs and it has not been conclusively proven that STMB vesicles, and micro- and nanoparticle levels are significantly raised in PE compared to normal pregnancies, and that these substances give rise to the inflammatory disorder seen in PE.

## Pro-angiogenic and anti-angiogenic factors in pre-eclampsia

## Pro-angiogenic factors, VEGF, PlGF and TGF-β

Vascular endothelial growth factor (VEGF) and platelet growth factor (PlGF) play a key role in placental angiogenesis and are believed to be secreted by trophoblast cells. VEGF is thought to be essential for integrity of the maternal endothelial cells.^37^ Both elevated and reduced levels of VEGF in the maternal circulation have been reported in PE.^38^ These conflicting results could be due to the methodologies used. Elevated levels could perhaps be due to the use of commercial kits that measure both the bound and the soluble forms of VEGF in the maternal circulation.

A longitudinal study showed that serum PlGF concentrations increased from 15–19 pg/ml through to 21–25 gestational weeks, and peaked at 27–30 weeks in uncomplicated pregnancies, in women with small-for-gestational-age (SGA) neonates and PE without SGA neonates, and thereafter the levels declined towards 35–36 gestational weeks.^39^ However, in PE complicated by SGA, the peak occurred at 21–25 gestational weeks, but at all times the levels were lower than in women with PE only.^39^

The transforming growth factor-β (TGF-β) family, especially TGF-β_1_ and TGF-β_3_, 40 have also been implicated in pre-eclampsia,^40^,^41^ but their exact mechanism of action is not known except to say that they are expressed in the pre-eclamptic placenta and reduce trophoblast proliferation, migration and invasion.^41^

## Anti-angionenic factors sFlt-1 and sEng

The anti-angiogenic factors are VEGF receptors (VEGFR1 and VEGFR2) and Eng. VEGFR1 is also known as fms-like tyrosine kinase-1 (Flt-1), which is membrane bound, while VEGFR2 is known as kinase insert domain receptor (KDR).^42^,^43^ It is known that sFlt-1, a spice variant of Flt-1, is the free form found in the circulation.^43^ Soluble Eng has anti-angiogenic effects, and as it has binding sites for TGF-β1 and β3,^44^ it is thought to play a role in PE.^45^

Venkatesha *et al.* found that Eng mRNA expression was significantly up-regulated in placental tissue (obtained at delivery), particularly in syncytiotrophoblasts in PE at 25 and 40 gestational weeks compared to age-matched control pregnancies.^44^ These researchers also found that this was accompanied by a significant rise in sera levels (obtained before delivery) of sEng in PE women compared to control pregnancies, and concluded that both sEng and sFlt-1 could be blocking the actions of TGF-β1 and VEGF, respectively. However, no significant differences in serum TGF-β1 levels were detected between normal-pregnancy and PE women.^44^

Venkatesha *et al.* further showed that administration of sEng to pregnant rats significantly increased the mean arterial pressure at 17–18 days of pregnancy but it had mild to modest effects on proteinuria. However, co-administration of sFlt-1 caused high levels of proteinuria, hypertension and evidence of the HELLP syndrome.^44^

## Imbalance in angiogenic and anti-angiogenic state in PE

There is increasing evidence that suggests an imbalance between pro-angiogenic and anti-angiogenic factors are responsible for the pathophysiological effects seen in PE,^46^,^47^ and these appear before clinical signs are apparent.^48^ However, it is not exactly known why some women develop PE while others with similar features, such as placental ischaemia and endothelial dysfunction, give birth only to SGA neonates without classical clinical signs of the disorder.^49^

Serum samples taken at the time of delivery have shown significantly increased sFlt-1 and decreased VEGF and PLGF concentrations in PE, compared to normotensive controls.^50^ *In vitro* studies showed that serum from PE inhibited tube formation in human umbilical vein endothelial cell (HUVEC) lines compared to that from controls, and administration of adenovirus expressing sFlt-1 to pregnant rats caused hypertension, albuminuria and glomerular endotheliosis, similar to that observed in PE.^50^

Cross-sectional studies conducted in our laboratories among black African women at term before delivery demonstrated that variations in plasma levels of pro-angiogenic (PlGF and TGF-β) and anti-angiogenic (sEng and sFlt-1) factors indicated an association with PE.^51^ In a similar study, we observed that serum sFlt-1 concentrations were significantly raised in early-onset PE and higher in late-onset PE compared to normotensive controls and chronic hypertensives, while VEGF was not detectable in all groups.^52^

A longitudinal study showed that patients with SGA neonates had significantly higher plasma sEng concentrations throughout their pregnancies, but in those who developed early- and late-onset PE, the levels were significantly higher at 23 and 30 gestational weeks, respectively, compared to normal pregnancies.^49^ In the case of plasma sFlt-1 levels, early- and late-onset PE had higher levels at 26 and 29 gestational weeks, respectively, compared to normal pregnancies.^49^ However, those with both early- and lateonset PE and those with SGA neonates had lower levels of PlGF throughout pregnancy, compared to controls.^49^ Other studies show similar findings.^53^,^54^

In addition, it was reported that plasma sFlt-1 levels were elevated in pre-eclamptics compared to normal pregnancies at 6–10 weeks and more so at 2–5 weeks prior to the development of a clinical diagnosis.^55^ A pilot study showed that extracorporeal removal of 17–34% of sFlt-1 from pre-eclamptic women between gestational ages 27 and 31 weeks lowered the blood pressure and reduced proteinuria and other complications.^56^

The disproportionate levels of anti-angiogenic factors such as sEng and sFlt-1, and pro-angiogenic factors such as VEGF, PlGF and TGFβ, are believed to cause generalised maternal endothelial dysfunctions, leading to hypertension, renal endotheliosis and blood coagulation.

## Immune factors and inflammation, cytokines and chemokines

There is increasing evidence suggesting that both innate and adaptive immune processes are involved in the pathogenesis of PE.^57^,^58^ Predominance of Th1 immunity is not only related to poor placentation but also to the exaggerated inflammatory response and endothelial dysfunction seen in PE.^59^

In a recent study it was shown that between 14 and 18 gestational weeks, serum tumour necrosis factor-α (TNF-α), interleukin 10 (IL-10) and interferon-γ (INF-γ) levels were significantly lower in PE than in normal pregnancy.^60^ In another study, it was shown that serum levels of circulating cytokines, IL-2, IL-4, IL-6, IL-8, IL-10, IL-12p40, IL-12p70, IL-18, INF-γ, TNF-α and chemokine interferon-γ-inducible protein (IP-10), monocyte chemotactic protein-1 (MCP-1) and adhesion molecules [intercellular adhesion molecule (ICAM-1) and vascular cell adhesion molecule (VCAM-1)] were raised in PE compared to controls.^58^

In early-onset PE, the plasma TNF-α and its receptors TNFR1, IL-1β and IL-12 levels, and heat shock protein-70 (Hsp-70) were significantly higher than in late-onset PE, while IL-10 concentrations were higher in late-onset than early-onset PE.^61^ Controversial findings have therefore been reported in the levels of some of the cytokines. The differences noted could have been due to the time of taking blood samples. For example, in the study by Kumar *et al.*,^60^ the samples were taken between 14 and 18 gestational weeks because 24 hours before delivery, raised levels of IL-4 and TNF-α were found, while the levels of INF-γ were not significantly different between those with PE and controls.^60^

However, it is believed that in PE compared to normal pregnancy, there is a shift to Th-1 type from Th-2 type of immunity. It is known that Th-1 type produces INF-γ and TNF-α, and hence it would be expected that the latter cytokines would be raised in the circulation.

A meta-analysis and a systematic review of published articles on concentrations of TNF-α, IL-6 and IL-10 in the maternal circulation showed that the concentrations were significantly higher in PE compared to controls.^63^ It is noteworthy that in one study in which TNF-α levels were measured, there was also no significant difference between PE patients and controls.^63^ From these data, Lau *et al.* concluded that in the third trimester, PE is associated with higher levels of TNF-α, IL-6 and IL-10 in the maternal circulation, compared to normal pregnancies, but they found insufficient evidence to state that this was so in the first and second trimesters as well.^62^

A recent study conducted at a mean gestational age of 34 weeks demonstrated that plasma IL-6, IL-8 and INF-γ levels were significantly higher in PE compared to age-matched normal pregnant and non-pregnant women. The level of TNF-α was not significantly different but the level of IL-10 was significantly higher in normotensives than pre-eclamptics.^63^ In addition, it was found that severe PE was associated with increased plasma levels of IL-8, IL-6, TNF-α, IL-12 and INF-γ, linking these cytokines with the exaggerated inflammatory response in this condition.^63^ Studies from our laboratories have just recently shown that blood levels of Th-1 (TNF-α, IL-2, IL-12p70), INF-γ and granulocytemacrophage colony-stimulating factor (GM-CSF), and Th-2 (IL-4, IL-5, IL-10 and IL-13) cytokines are similar in PE and normotensive pregnant women.^64^

## Low oxygen tension, oxidative stress in gene expression levels in PE

In early-onset PE, oxidative stress caused by low oxygen tension or by disruption of the oxygen-sensing mechanism in placentas is believed to cause over-expression of hypoxia inducible factor-1 (HIF-1α) in placental tissue, and also to the release of increased levels into the circulation.^65^ In normal pregnancy, placental expression and formation of HIF-1α increased in a hypoxic environment during the first trimester and this was paralleled by TGF-β_3_, of which early trophoblast differentiation and placental expression of both molecules remained high until about the 10th gestational week when placental O_2_ levels began to increase.^66^ This was speculated to be responsible for extravillous trophoblast (EVT) outgrowth and invasion of the spiral arteries. However, it was noted that in PE the expression and formation of HIF-1α and consequently TGF-β_3_ remained high, resulting in shallow trophoblast invasion of the spiral arteries.^66^ These findings were confirmed by other researchers.

Increased expression of the haeme (Hb) gene in the presence of hypoxia or oxidative stress has also been noted in PE placentas, and together with foetal haemoglobin (HbF), is thought to be involved in the pathogenesis of PE.^33^ In a review article, Hansson *et al.* in 2014 showed that free Hb, in addition to causing oxidative stress, also caused placental and kidney damage.^33^

Placental hypoxia and perhaps oxidative stress, which occurs in PE, is also known to upregulate the gene expression and formation of Eng in placental tissue, perhaps via TGF-β_3_.^67^,^68^ The action of Eng and its receptor sEng have already been discussed. Perhaps in a similar manner, there is over-expression of genes responsible for the formation of sFlt-1, PlGF and VEGF in PE placentas.^65^,^68^ However, it has to be noted that for ethical reasons, it is difficult to study gene expression in placentas prior to actual clinical diagnosis of PE.

## The RAAS and angiotensin II AT -1 receptorauto-antibodies

In a recent review article Verdonk *et al.* presented a detailed account of the involvement of RAAS and Ang II AT-1 receptor auto-antibodies (AT-1AA) in the pathophysiology of pre-eclampsia.^10^ Readers are advised to refer to Verdonk *et al.*^10^ for details. They stated that in normal pregnancies, particularly in the early stages of gestation, there is an increase in maternal blood volume and a decrease in total resistance, and to counteract a fall in blood pressure, the RAAS is activated, resulting in sodium and water retention. However, in PE in contrast to normal pregnancy, the intravascular blood volume and cardiac output are reduced, while the total peripheral resistance is increased, and most components of the RAAS are downregulated.^10^

These findings led them to conclude that in pre-eclampsia, the suppression of most components of the RAAS could lead to increased response to Ang II and AT-1AA. They reported that the exact role of the RAAS and AT-1AA systems in PE remains unanswered, suffice to state that the sensitivity of Ang II receptors to Ang II is increased, and angiotensinogen synthesis is stimulated by high circulatory oestrogen levels in the first 10 weeks of pregnancy.^10^

High-molecular weight angiotensinogen levels were found to be about 25% higher than total angiotensinogen levels in PE, compared to 16% in normal pregnancy.^69^ However, it has been found that plasma renin activity, Ang II and aldosterone levels were decreased.^70^ At present, evidence of the exact role of the RAAS in PE is therefore lacking.

Circulating auto-antibodies to AT-1AA have been shown to increase after 20 weeks of gestation.^71^ Others have shown that AT-1AA was more predictive in late-onset than in early-onset PE.72 It is possible that Ang II, by activating the AT-1 receptors on human trophoblasts, could play a role in shallow trophoblast invasion of the spiral arteries through secretion of plasminogen activator inhibitor-1 (PAI-1). Similar findings for AT-1AA were noticed in *in vitro* studies using human mesangial cells, where it caused increased secretion of PAI-1 and IL-6, compared to IgG from normotensive patients.^73^ It was further speculated that the latter actions of AT-1AA could account for the renal damage seen in PE patients.^73^

Xia and Kellems have presented a detailed review on the pathophysiological role of AT-1AA.^74^ They have shown that that these auto-antibodies play a critical role in PE, and blockade of AT-1 receptors in animal models reversed the signs and symptoms of PE by reducing the circulatory levels of sFlt-1 and IL-6.^74^

However, it remains to be shown conclusively that in human patients, AT-1AA plays an important role in the pathophysiology of PE, since most of the experiments were conducted in animal models, which may not represent what happens in PE. In addition, it has not been conclusively shown that in every pre-eclamptic woman, the levels of AT-1AA are raised.

## Hydrogen sulphide

Hydrogen sulfide (H_2_S) is a gaseous signalling molecule in humans and animals. It is produced in endothelial cells.^75^ It has vaso-relaxant properties and is involved in uterine contractility.^76^,^77^ Endogenously produced H_2_S also has angiogenic^75^ and antiinflammatory properties.^75^,^78^ In the latter case, H_2_S acts at the endothelial–leukocyte interface.^78^ Chronic administration of H_2_S was found to have hypotensive effects in a rat model and reduced infarct in ischaemic–reperfusion injury in experimental rats.^79^

The production of H_2_S requires one of two enzymes: cystathionine γ-lyase (CSE) or cystathionine β-synthase (CBS).^80^ Both these enzymes are localised in foetal endothelial cells of both the stem and chorionic villi, and the Hofbauer cells express CBS mRNA.^80^

In early-onset but not late-onset PE, CBS mRNA expression was down-regulated.^80^ A recent study showed that mRNA expression of CSE was reduced in pre-eclamptic placental tissue and in women with SGA neonates, compared with normal pregnancy.^81^ The reduction in CSE expression was accompanied by reduction in the concentration of H_2_S in the maternal circulation.^81^ In addition, it was found that trophoblasts and mesenchymal cells in the core of the chorionic villi were the sites for expression of CSE.^81^

Inhibition of CSE by DL-propargylglycine (PAG) in pregnant mice resulted in hypertension and elevation in sFlt-1 and sEng levels in the circulation, and also caused placental abnormalities, while administration of GYY4137, which inhibits the action of PAG, reduced the levels of circulating sFlt-1 and sEng and restored foetal growth.^81^ This illustrates that H_2_S is required for placental development.

Furthermore, it was also shown in *in vitro* studies that dysregulation of the CSE/H2S pathway affected spiral artery remodelling and placental development.^81^ In addition, it was found that inhibition of CSE with PAG in placental explants taken from first-trimester pregnancies reduced PlGF production. Wang *et al.* are of the view that their findings imply that endogenous H_2_S is required for placental development and foetal and maternal well-being.^81^ These findings perhaps show that H_2_S plays a role in the pathogenesis and pathophysiology of PE. However, it is felt that plasma H_2_S levels were overestimated in some of the above studies and may not reflect the true values.^82^

Nulliparity has been suggested as a risk factor for PE.^83^ The risk of pre-eclampsia was 26% in nulliparous patients versus 17% in parous [RR and 95% CI: 1.5 (1.3–1.8)] subjects. The risk of PE is also increased with a history of abortion and changed paternity. There seems to be a genetic component. Both mother and foetus contribute to the risk of PE, the contribution of the foetus being affected by paternal genes. An immune-based pathology is also proposed, whereby prolonged exposure to foetal antigens protects against PE in a subsequent pregnancy with the same father.^84^,^85^ Finally, a reason why PE is more common in nulliparous than multiparous women could be that in the latter, the uterine and spiral arteries develop a larger bore, which is easier for trophoblastic invasion.^83^

## Conclusion

The exact aetiology of PE remains elusive but much of the pathophysiology has been explained. The current theory is one of balance between angiogenic and anti-angiogenic factors. Measurement of circulatory angiogenic and anti-angiogenic proteins as biomarkers could possibly indicate placental dysfunction and differentiate PE from other disorders, such as gestational hypertension and chronic glomerulonephritis. In addition, biomarkers such as those stated above are reproducible, linked to the disease, and above all, are easy to interpret.

**Fig. 1.**
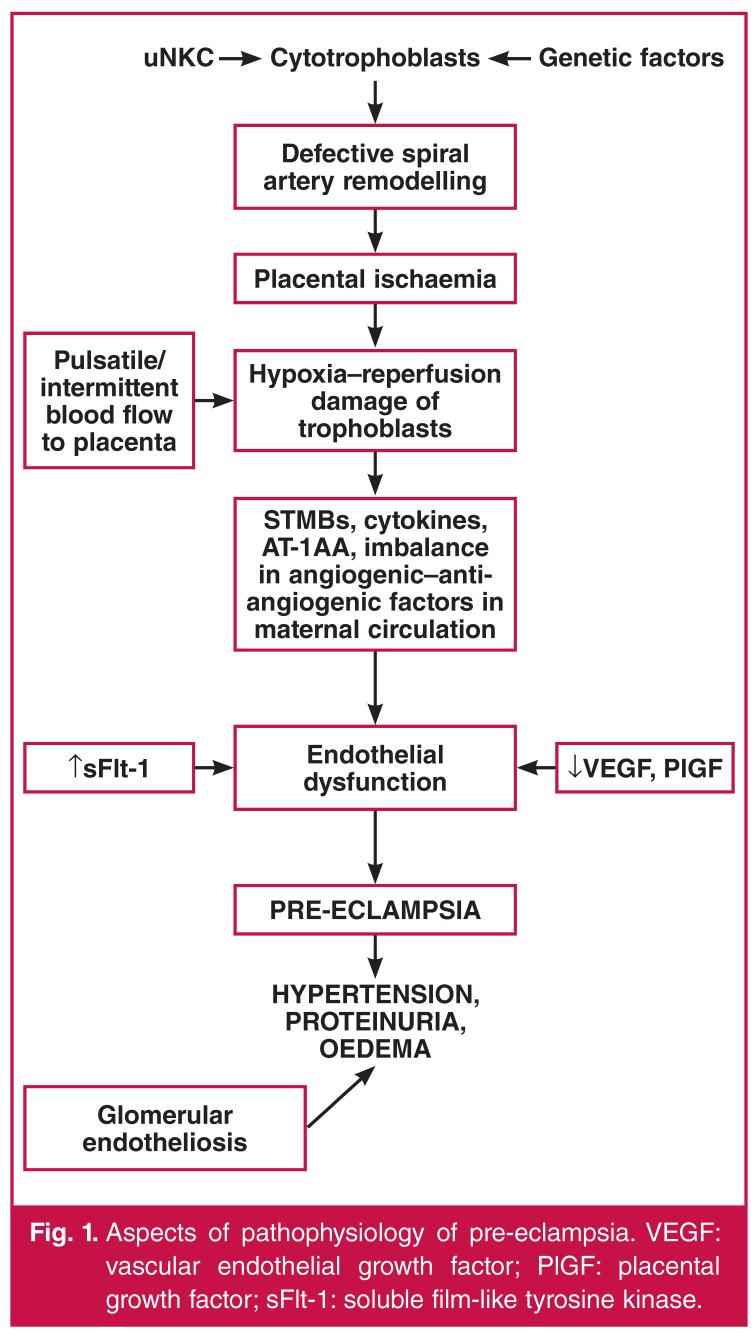
Aspects of pathophysiology of pre-eclampsia. VEGF: vascular endothelial growth factor; PlGF: placental growth factor; sFlt-1: soluble film-like tyrosine kinase.

See Fig. 1 for some aspects of the pathophysiology of pre-eclampsia.

**Key messages:**